# Linked Selection and Gene Density Shape Genome‐Wide Patterns of Diversification in Peatmosses

**DOI:** 10.1111/eva.13767

**Published:** 2024-08-19

**Authors:** Olena Meleshko, Michael D. Martin, Kjell Ivar Flatberg, Hans K. Stenøien, Thorfinn Sand Korneliussen, Péter Szövényi, Kristian Hassel

**Affiliations:** ^1^ Department of Natural History, NTNU University Museum Norwegian University of Science and Technology Trondheim Norway; ^2^ Section for GeoGenetics, Globe Institute University of Copenhagen Copenhagen Denmark; ^3^ Department of Systematic and Evolutionary Botany & Zurich‐Basel Plant Science Center University of Zurich Zurich Switzerland

**Keywords:** gene density, genome evolution, introgression, linked selection, peatmoss, speciation

## Abstract

Genome evolution under speciation is poorly understood in nonmodel and nonvascular plants, such as bryophytes—the largest group of nonvascular land plants. Their genomes are structurally different from angiosperms and likely subjected to stronger linked selection pressure, which may have profound consequences on genome evolution in diversifying lineages, even more so when their genome architecture is conserved. We use the highly diverse, rapidly radiated group of peatmosses (*Sphagnum*) to characterize the processes affecting genome diversification in bryophytes. Using whole‐genome sequencing data from populations of 12 species sampled at different phylogenetic and geographical scales, we describe high correlation of the genomic landscapes of differentiation, divergence, and diversity in *Sphagnum*. Coupled with evidence from the patterns of covariation among different measures of genetic diversity, phylogenetic discordance, and gene density, this provides strong support that peatmoss genome evolution has been shaped by the long‐term effects of linked selection, constrained by distribution of selection targets in the genome. Thus, peatmosses join the growing number of animal and plant groups where functional features of the genome, such as gene density, and linked selection drive genome evolution along predetermined and highly similar routes in different species. Our findings demonstrate the great potential of bryophytes for studying the genomics of speciation and highlight the urgent need to expand the genomic resources in this remarkable group of plants.

## 1 Introduction

Since the development of next‐generation sequencing technologies, the field of speciation genomics has been thriving, and our knowledge about speciation, one of the most fundamental processes in evolution, has widened drastically (Seehausen et al. 2014; Wolf and Ellegren 2017; Jiggins 2019). Deep insights into ongoing and past speciation processes can be achieved by exploring interspecific genetic differentiation and its genome‐wide distribution (Wolf and Ellegren 2017). We now know that patterns of differentiation between species are highly variable across the genome (see Seehausen et al. 2014 for a review). This is interpreted as a result of speciation by gradual establishment of barriers to gene flow due to divergent selection associated with regions of accentuated differentiation, that is, differentiation islands (Wu 2001; Turner, Hahn, and Nuzhdin 2005; Feder and Nosil 2010). However, the heterogeneous differentiation landscape may have little to do with speciation itself, but rather be derived from variation in recombination rate and distribution of functional features of the genome, that is, genome architecture, that in turn define the effects of selection or gene flow across the genome (Nachman and Payseur 2012; Cruickshank and Hahn 2014; Jiggins 2019). Positive and purifying selection lead to a decrease in genetic diversity and effective population sizes (*N*_e_) at sites linked to the actual target of selection due to the process of genetic hitchhiking (Smith and Haigh 1974; Charlesworth, Morgan, and Charlesworth 1993). These polymorphism‐reducing effects are termed linked selection (reviewed by Cutter and Payseur 2013), and result in a local increase in among‐species differentiation intensified in regions with low recombination and high density of functional elements (Kaplan, Hudson, and Langley 1989; Begun and Aquadro 1992; Payseur and Nachman 2002). Long‐term linked selection will also locally decrease divergence due to repeated reduction in variation at linked sites (Cruickshank and Hahn 2014; Burri et al. 2015).

According to the linked selection model, the distribution of genetic differentiation can be conjectured by variation in genome architecture (Charlesworth 1998; Nachman and Payseur 2012; Cruickshank and Hahn 2014). When genome architecture is highly conserved across species, diversifying lineages would be experiencing similar outcomes of selection, leading to highly similar genome‐wide distributions of differentiation in independent species across a speciation continuum (Cutter and Payseur 2013; Burri 2017). Such highly correlated heterogeneous differentiation landscapes have been described in various groups of birds (Burri et al. 2015; Van Doren et al. 2017; Han et al. 2017; Delmore et al. 2018), insects (Martin et al. 2013; Kronforst et al. 2013; Edelman et al. 2019) and vascular plants (Renaut et al. 2013; Stankowski et al. 2019).

Genome evolution has been studied more and understood better in crops or model plants, while nonmodel plants still lack extensive genomic resources and hence understanding of how their genomes evolve in the process of speciation. This topic has hardly been investigated at all in nonvascular plants, such as bryophytes, where genomic resources are nonexistent in the vast majority of species. Bryophytes are the largest group of nonvascular land plants and share a common ancestor with all vascular plants (Puttick et al. 2018). Bryophytes play a crucial role in the global carbon and nitrogen cycling, as well as in ecosystems succession, and provide a wide range of important ecosystem services (reviewed in Turetsky 2003; Alatalo et al. 2020). These early land plants represent hundreds of millions of years of evolutionary history encompassing major evolutionary transitions after land colonization (reviewed by Fernandez‐Pozo et al. 2022). The key feature differentiating these plants from angiosperms is that haploid gametophyte dominates their life cycle, which makes them more efficient in purging deleterious mutations, even more so in predominantly selfing species (Szövényi et al. 2014). Bryophytes have high incidences of clonal reproduction (Stenøien and Såstad 2001; Cronberg, Rydgren, and Økland 2006) which should lower their effective recombination rate. In contrast to angiosperm genomes where chromosomes have distinct gene‐rich regions at the ends and gene‐sparse regions at the center, functional elements are distributed evenly along the chromosomes in genomes of bryophytes (Lang et al. 2018; Diop et al. 2020; Li et al. 2020; Szövényi, Gunadi, and Li 2021; Healey et al. 2023). These features of bryophyte biology could imply that strong effects of linked selection might be observed in their genomes.

One of the emerging model groups in bryophyte genomics is peatmosses (*Sphagnum* L., Sphagnaceae), a relatively well‐studied, compared with the rest of bryophytes, genus comprising five subgenera with numerous species with wide geographical distributions (Michaelis 2019). We know that most peatmoss species are haploid, with a small and surprisingly stable genome size (0.39–0.49 pg DNA, Temsch, Greilhuber, and Krisai 1998), chromosome number (*n* = 19) and karyotype (Fritsch 1991) across species. A recent study shows that there is high collinearity between the genomes of two peatmosses *Sphagnum divinum* and *S. angustifolium* (Healey et al. 2023) which represent different subgenera of peatmosses. These traits enable side‐by‐side comparison of multiple species with a speciation continuum, which allows using peatmosses to study bryophyte genome evolution. They also indicate that genome structure may be conserved in peatmosses which would exacerbate the effects of linked selection. If this is the case, genomic differentiation landscapes are expected to be similar across multiple species (Cutter and Payseur 2013; Burri 2017), and variation in this landscape should be associated with variation in genetic diversity and distribution of selection targets in the genome (Delmore et al. 2018; Rettelbach, Nater, and Ellegren 2019). Peatmosses are also characterized by relatively large *N*_e_ (Stenøien and Såstad 1999; Szövényi et al. 2008), ancient and recent interspecific hybridization, and widespread incomplete lineage sorting (ILS) stemming from rapid radiation of the genus (Meleshko et al. 2021) which occurred 7–20 Ma (Shaw et al. 2010). Therefore, the effects of gene flow and ILS might result in patterns of co‐distribution of genome‐wide landscapes of genetic diversity, differentiation, and divergence that differ from that produced by linked selection (Wolf and Ellegren 2017; Liang et al. 2022).

In addition to being a model of bryophyte genomics, *Sphagnum* is the most important plant genus for global carbon cycling because it is a key component of peatland ecosystems. The amount of carbon stored in peatlands exceeds that stored in all other types of vegetation combined even though peatlands only occupy ca. 3% of terrestrial area worldwide (Yu et al. 2011; IUCN 2021). Peatmoss distribution and species diversity are currently concentrated in the Northern Hemisphere (Laine et al. 2018), which is warming up faster than the global average (reviewed in Rantanen et al. 2022). This is especially concerning given that peatlands turn from carbon sink into source when destabilized or overheated (Frolking et al. 2011; Wilson et al. 2016). Studying speciation processes underlying peatmoss diversity may provide important insights which can help conservation efforts and mitigate the effects of climate change on peatmoss‐mediated carbon cycling.

In this study, we use peatmosses to characterize the processes affecting genome diversification in bryophytes. We use low‐depth whole‐genome sequencing data from 12 peatmoss species (Meleshko et al. 2021) to describe and compare the genomic landscapes of differentiation, divergence, and diversity across the speciation continuum in *Sphagnum*. The selected species are co‐occurring haploid species that represent all five subgenera within the genus and were sampled at different phylogenetic and geographical scales. The species all evolved in the second wave of diversification and are estimated to be younger than 14 Ma (Shaw et al. 2010). Specifically, we aim to (i) estimate genome‐wide distributions of genetic differentiation, divergence, and diversity in all species and pairwise species comparisons, (ii) evaluate the degree of their similarity across the species and species comparisons, (iii) explore how these statistics co‐vary when taking interspecific variability into account, and (iv) estimate the relationship between these statistics and genome‐wide distribution of phylogenetic discordance and density of selection targets. We use these analyses to identify the factors shaping the genomic landscape of diversification in this diverse group of plants, seeking to differentiate between the effects of linked selection, gene flow variation, and ILS.

## 2 Results

### 2.1 Study Species, Sampling, and Sequencing

**FIGURE 1:**
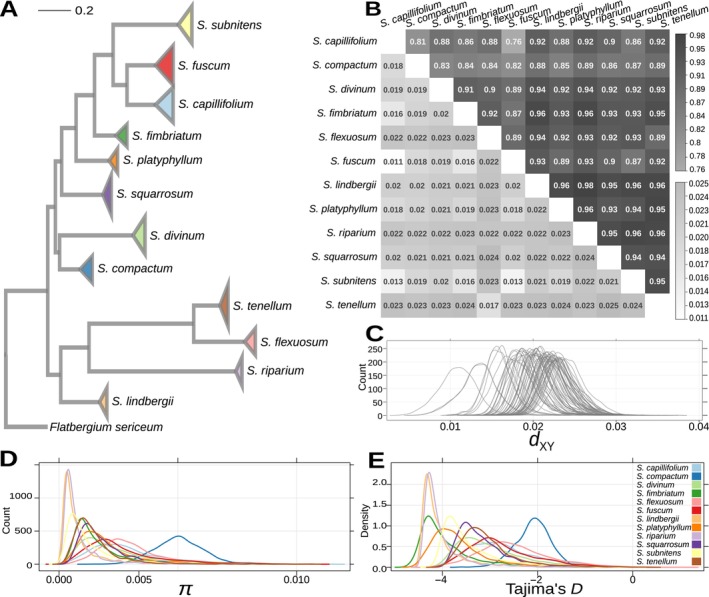
Genetic diversity and differentiation. (A) Phylogenetic relationships among the studied species as reconstructed by Meleshko et al. (2021), with all the branch tips within each species collapsed as shown by triangular symbols that cover the same space as the clade. Colors represent species as shown in (E). (B) Pairwise *d*_XY_ (lower diagonal) and *F*_ST_ (upper diagonal) for all species pairs. The colors represent the value of the corresponding statistics as shown on the right: Lower scale bar—*d*_XY_, upper scale bar—*F*_ST_. (C) Distribution of pairwise *d*_XY_ in 100‐kbp sliding windows for all species pairs. (D) Distribution of nucleotide diversity (𝜋), and (E) Tajima's *D* in 100‐kbp sliding windows for all species, colors represent species as shown on the right in (E).

We obtained previously published (Meleshko et al. 2021) whole‐genome resequencing data which include 190 individuals from sympatric/parapatric and allopatric populations of 12 peatmoss species (Figure 5A, Table S1). Phylogenetic relationships among the species are represented by the species tree (Figure 1A) reconstructed by (Meleshko et al. 2021). For each of the species, at least one population from each of the three geographical locations (Norway, Germany, and Austria) was sampled, with one to four individuals included in each population (Table 1). The average per‐sample number of raw sequencing reads after quality filtering was 65 ± 45 M (SD) reads, which were mapped to a reference genome assembly of *Sphagnum angustifolium* v0.5 (Healey et al. 2023). The average per‐sample portion of uniquely mapped reads was 38 ± 16% (SD) and did not differ significantly among the species in line with the previous findings, reflecting the variation in per‐sample endogenous DNA content (Meleshko et al. 2021). The sequencing coverage of 6.25 ± 2.6 (SD) was obtained after mapping of reads.

**TABLE 1:** Sampling summary.

Species	Subgenus	Population							Total
		Austria			Norway			Germany	
		A1	A2	Total	N1	N2	Total		
*S. capillifolium*	*Acutifolia*	6	1	7	3	5	8	2	17
*S. compactum*	*Rigida*	3	0	3	3	5	8	2	13
*S. divinum*	*Sphagnum*	6	3	9	3	6	9	0	18
*S. fimbriatum*	*Acutifolia*	0	4	4	4	0	4	3	11
*S. flexuosum*	*Cuspidata*	0	3	3	3	6	9	2	14
*S. fuscum*	*Acutifolia*	6	3	9	3	5	8	2	19
*S. lindbergii*	*Cuspidata*	3	0	3	3	6	9	2	14
*S. platyphyllum*	*Subsecunda*	1	3	4	3	5	8	0	12
*S. riparium*	*Cuspidata*	3	2	5	3	5	8	1	14
*S. squarrosum*	*Acutifolia*	3	5	8	3	6	9	2	19
*S. subnitens*	*Acutifolia*	3	6	9	3	5	8	2	19
*S. tenellum*	*Cuspidata*	0	6	6	3	9	12	2	20

*Note:* Number of samples collected from each of the populations for each of the 12 studied *Sphagnum* species: A1—Tamsweg district, Austria; A2—Upper Austria, Austria; N1—Namsos area, Norway; N2—Trondheim area, Norway; G—Germany.

### 2.2 Genomic Variation and Divergence Across Peatmosses

To describe genetic variability, among‐species divergence and differentiation, we first calculated the genome‐wide average of within‐population nucleotide diversity (𝜋) and Tajima's *D* for all species, as well as among‐species genetic diversity (*d*_XY_) for all species pairs, based on biallelic sites in 1774 100‐kbp nonoverlapping windows across all genomic scaffolds longer than 2 Mbp (49 scaffolds, in total equal to 44.3% of the total length of the reference genome). We also estimated the genome‐wide weighted pairwise genetic differentiation (*F*_ST_), as well as pairwise *F*_ST_ in the same 100‐kbp windows, for all species pairs. We found that genome‐wide estimates of 𝜋 varied between 0.0008 and 0.005 per species (0.0022 ± 0.0012 SD), indicating moderate within‐population diversity (Leffler et al. 2012; Carvalho et al. 2019). In turn, average pairwise *d*_XY_ ranged from 0.011 to 0.025 (0.021 ± 0.0027 SD) (Figure 1B), corresponding to high levels of sequence divergence among species (Han et al. 2017; Stankowski et al. 2019), which is in line with the strong genetic differentiation among species inferred by genome‐wide *F*_ST_ (0.75–0.98, 0.91 ± 0.04 SD, Figure 1B). In peatmosses, high levels of *F*_ST_ are normally observed even among sister species (e.g., Yousefi et al. 2017; Petlund 2021) and among populations of the same species (e.g., Yousefi et al. 2019; Nilsen 2021).

**FIGURE 2:**
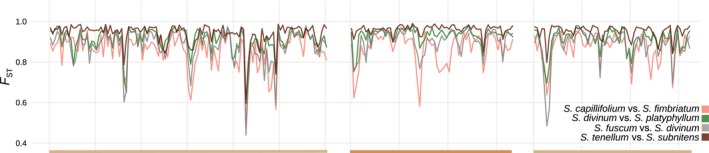
Pairwise differentiation (*F*_ST_) in 100‐bp sliding windows across the three longest scaffolds (*x* axis, designated by colors) in four arbitrary species pairs involving seven species. Colors correspond to pairwise comparisons as shown on the right.

The sliding‐window based estimates permitted us to assess the genome‐wide distribution of the statistics. We found that per‐window estimates of 𝜋 and Tajima's *D* in the investigated species varied from 0.00008 to 0.011 (0.0022 ± 0.0017 SD) (Figure 1D) and from −4.71 to 0.96 (−3.19 ± 0.85 SD) (Figure 1E), respectively, whereas in different pairwise comparisons, *F*_ST_ ranged from 0.17 to 0.99 (0.90 ± 0.01 SD) and *d*_XY_ ranged from 0.004 to 0.037 (0.021 ± 0.0033 × 10^−3^ SD) (Figure 1C). We found that in all pairwise comparisons, *F*_ST_‐based differentiation was very high along most of the scaffolds interspersed with 2–5 narrow *F*_ST_ valleys indicating localized differentiation that is lower than the genome‐wide average (Figure 2). Within‐species nucleotide diversity (𝜋) and Tajima's *D*, as well as divergence (*d*_XY_), followed the opposite pattern, with peaks coinciding with the *F*_ST_ valleys. This resembles the pattern discovered in studies describing the late stages of speciation, where a small number of less differentiated genomic islands are present in an otherwise highly differentiated landscape (Riesch et al. 2017; Han et al. 2017; Ravinet et al. 2018). This initial visual assessment revealed that variation of *F*_ST_, *d*_XY_, and 𝜋 across the genome tends to be correlated among species. The remarkable similarity in the distribution of pairwise *F*_ST_ values is shown for four randomly selected species pairs in Figure 2. Such a correlation would strongly suggest that evolution of genome diversification in peatmosses is indeed constrained by one or more genomic features conserved across species leading to similar outcomes of selection and/or introgression in different species (Cutter and Payseur 2013; Burri et al. 2015). To further investigate this question, we carried out various analyses to verify and describe the correlated nature of genome‐wide genetic diversity and differentiation, which we provide below.

### 2.3 Correlated Landscape of Differentiation and Diversity Across the Peatmoss Species

**FIGURE 3:**
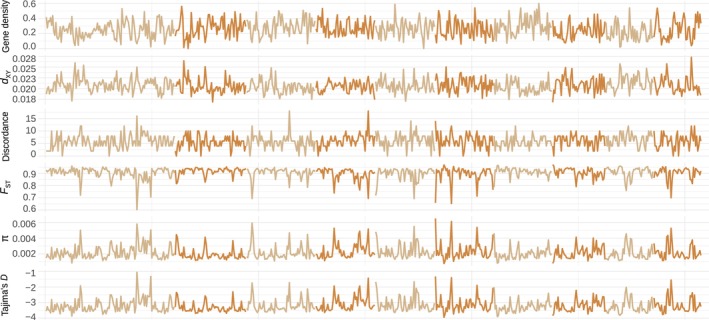
Correlated genomic landscapes of summary statistics (the mean window‐based values) in 100‐kbp sliding windows across the 10 longest scaffolds (*x* axis). Alternating colors represent different scaffolds.

In order to summarize and normalize the level of differentiation and diversity across all species pairs and species in one statistic and to describe the strength of correlation among species, we carried out a principal component analysis (PCA) on all statistics calculated. Using this approach, the correlation among the differentiation landscapes in different species would be reflected by the proportion of variance explained by the main component, and the loadings on the main component would represent a single relative differentiation landscape for the group (Burri 2017). We performed a PCA on the sliding window data for 66 among‐species estimates of pairwise *F*_ST_ and *d*_XY_, and for 12 within‐species estimates of 𝜋 and Tajima's *D*. For each of these statistics, the genomic landscapes were highly correlated across the species or pairwise comparisons, with most of the variation explained by the first principal component (PC1) (73%, 73%, 56%, and 57% for *F*_ST_, *d*_XY_, 𝜋, and Tajima's *D*, respectively, Figures S1A,B and S2A,B). For each statistic, the loadings on PC1 were strongly and significantly (*p* < 0.0001) correlated with corresponding window‐based values in all individual comparisons (Figure S1C), and with the mean window‐based values, that is, per‐window value averaged among all species or pairwise comparisons (*r*_S_ > |0.99|, *p* < 0.0001, Figure S2D,E). In turn, the mean window‐based values were highly significantly (*p* < 0.0001, Benjamini and Hochberg correction for multiple tests) correlated with corresponding window‐based values in each species or pairwise comparison for each statistic. Since the mean window‐based values are easier to interpret than the loadings on PC1, we used them as a representation of the genomic landscape for the corresponding statistic in the subsequent analyses (Table S2). Altogether, our analyses revealed highly correlated landscapes of diversity, divergence, and differentiation among the investigated peatmoss species (Figure 3, Figure S3).

### 2.4 The Effects of Recurrent Linked Selection

The high correlations among the landscapes that we observed suggest that the evolution of genome diversification in peatmosses might be constrained by genomic features conserved across the species, which results in similar effects of selection (direct or indirect), introgression, or ILS in different species (Cutter and Payseur 2013; Burri 2017). To further dissect the relative contributions of these processes to the evolution of the diversification landscapes in peatmosses, we assessed the spatial distribution of these statistics and their correlation across the genome while taking among‐species variability into account.

To do so, and to better describe how these population genetic statistics co‐vary, we calculated the Spearman‐rank correlation among mean window‐based values of gene density, 𝜋, Tajima's *D*, *d*_XY_, and *F*_ST_. We also investigated if selected genomic features could account for most of the variability in the genomic landscapes we observed. Density of functional elements, such as genes, is one of the main features of genome architecture that modulates the effect of evolutionary forces on the genomic pattern of diversification and divergence (Phung, Huber, and Lohmueller 2016; Schrider 2020). Gene density can predict variation in genetic diversity (Flowers et al. 2012) and has been previously shown to be one of the major drivers of evolution of correlated genomic landscapes via linked selection in other species (Burri et al. 2015; Delmore et al. 2018; Stankowski et al. 2019). Gene density was calculated in 100‐kbp sliding windows as the ratio of the total number of bases within gene regions in a sliding window to the total length of the window (Figure S4). We also calculated gene count as the total number of genes per sliding window.

**FIGURE 4:**
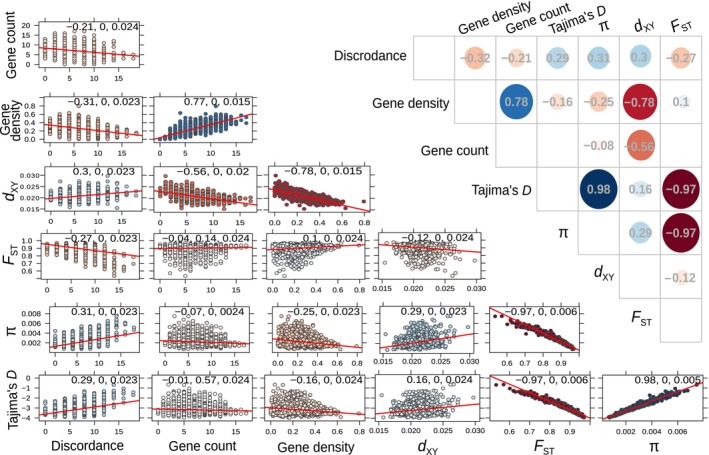
Correlation structure of the summary statistics investigated. The numbers on the scatterplots correspond to Spearman's rank correlation coefficients followed by probability values adjusted for multiple tests and standard error. Tajima's *D*, 𝜋, *d*_XY_, and *F*_ST_ refer to mean window‐based Tajima's *D*, 𝜋, *d*_XY_, and *F*_ST_, respectively. Solid red lines represent the linear regression between the corresponding statistics. The color and size of circles in the upper panel represent the strength of correlations, with blue color representing positive correlation, and red color—negative correlation.

First of all, we found strong negative correlations between *F*_ST_ and 𝜋, and between *F*_ST_ and Tajima's *D* (*r*_S_ = −0.97, *p* < 0.0001, Figure 4). This suggests that *F*_ST_ valleys are associated with more shared polymorphisms and a higher proportion of intermediate frequency variants, which can potentially be caused by the effect of selection target density on the strengths of gene flow, rate of sorting of ancestral polymorphisms, or positive/negative selection. Assuming recurrent linked selection, gene density is expected to be positively correlated with genetic differentiation and negatively correlated with within‐species genetic diversity (Cutter and Payseur 2013). Indeed, we found that gene density was weakly positively correlated with *F*_ST_ and negatively correlated with 𝜋 (*r*_S_ = 0.1 and *r*_S_ = −0.25, respectively, *p* < 0.0001, Figure 4). By repeatedly reducing nucleotide diversity close to the target regions, recurrent positive or purifying selection should also create local reductions of *d*_XY_ (Burri et al. 2015; Matthey‐Doret and Whitlock 2019), which is the pattern we observe. Indeed, *d*_XY_ was positively correlated with 𝜋 and weakly negatively with *F*_ST_ (*r*_S_ = 0.29 and *r*_S_ = −0.12, respectively, *p* < 0.0001, Figure 4). High values of *F*_ST_ saturated around 1 could explain lower strength of correlations of *F*_ST_ with other statistics. We found a stronger skew toward an excess of rare alleles (as expressed by negative Tajima's *D*) in regions with higher gene density (*r*_S_ = −0.16, *p* < 0.0001, Figure 4). These findings strongly suggest that the differentiation landscape has been shaped by recurrent linked selection.

### 2.5 Gene Flow and the Differentiation Landscape

**FIGURE 5:**
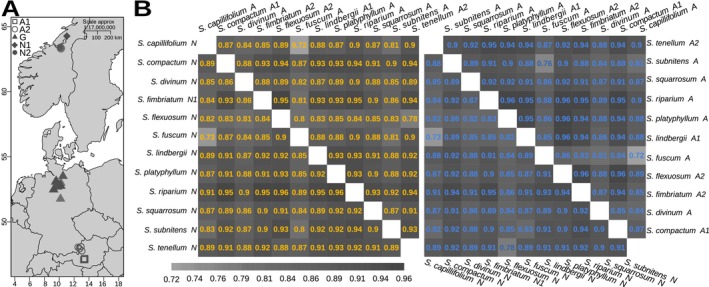
Genetic differentiation in sympatric and allopatric comparisons. (A) Geographic locations of the sampled populations: A1—Tamsweg district, Austria; A2—Upper Austria, Austria; N1—Namsos area, Norway; N2—Trondheim area, Norway; G—Germany. The symbols represent sampling locations as shown on the left. (B) Pairwise *F*_ST_ among sympatric (in blue) and allopatric (in orange) populations in all species pairs. The plot on the right shows sympatric populations from Norway (lower diagonal) and Austria (upper diagonal). Population codes: A1—Tamsweg district, Austria; A2—Upper Austria; A—all from Austria combined; N1—Namsos area, Norway; N2—Trondheim area, Norway; N—all from Norway combined.

**FIGURE 6:**
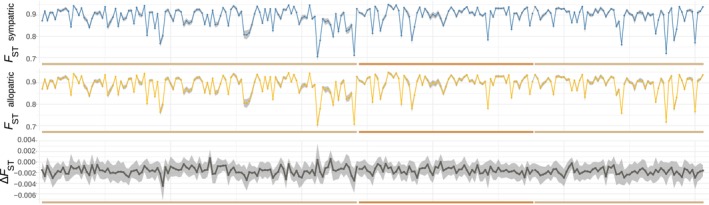
Mean per‐window pairwise *F*_ST_ in sympatric (in blue) and allopatric (in orange) populations across the three longest scaffolds (*x* axis, designated by colors, same order as on Figure 2). The bottom panel shows the mean per‐window difference between allopatric and pairwise sympatric *F*_ST_ for each pair; light‐gray polygons correspond to confidence intervals.

Alternatively, the regions with accentuated differentiation might have occurred when reproductive barriers formed in the process of speciation leading to locally reduced gene flow (Cutter and Payseur 2013; Burri et al. 2015; Ma et al. 2018). In such a case, however, both *F*_ST_ and *d*_XY_ are expected to be elevated in these regions relative to the rest of the genome (homogenized by gene flow), which does not correspond to our findings. Despite the extensive interspecific hybridization among co‐occurring peatmoss species, postzygotic barriers seem to have been established in the studied species, leading to very low levels of recent, postspeciation interspecific gene flow (Meleshko 2021). Nevertheless, even low levels of gene flow can have a profound effect on genome evolution by reinforcing reproductive isolation among well‐separated species (Hopkins 2013; Twyford, Kidner, and Ennos 2015). Assuming the differentiation landscape is affected by gene flow, one could expect to see a consistent difference between sympatric and allopatric comparisons (Burri et al. 2015; Yamasaki et al. 2020). Therefore, we tested if closely located populations of each species pair are less or more differentiated than their allopatric populations, which would suggest that gene flow is confined to or hampered in sympatric populations (Renaut et al. 2013; Martin et al. 2013). We calculated *F*_ST_ among sympatric and allopatric populations sampled in Norway and Austria for all species pairs (Figure 5B) and found no significant correlation between genetic differentiation and geographical distance between species (Mantel test, *r* = −0.08, *p* = 0.88). We then calculated the difference between mean *F*_ST_ in sympatric comparisons and mean *F*_ST_ in allopatric comparisons in 100‐kbp sliding windows for each species pair (Figure 6) and performed a PCA to identify if variation in distribution of this difference along the genome is correlated among the comparisons in the same way as the overall interspecific *F*_ST_. We found that only 14.6% of variation was explained by *PC1* (Figure S5), suggesting that there was no systematic difference between allopatric and sympatric comparisons. These findings rule out recent gene flow as a major factor affecting the differentiation landscape. We also found that *d*_XY_ was strongly negatively correlated with gene density (*r*_S_ = −0.78, *p* < 0.0001, Figure 4), which provides further support for our recurrent linked selection scenario (Han et al. 2017). Taken together, the findings suggest reduced impact of linked selection or efficacy of selection acting in the *F*_ST_ valleys and indicate that recurrent linked selection is one of the main forces that contributes to the correlated pattern of genomic divergence, differentiation, and diversity observed.

### 2.6 The Effect of the Stochastic Coalescent Processes

In our previous study, we found that ILS and not introgression was the primary factor causing extensive genome‐wide phylogenetic discordance among the investigated peatmoss species (Meleshko et al. 2021). Therefore, one might expect that, besides recurrent selection, ILS might also have influenced the genomic landscape of differentiation. In the presence of positive or purifying selection, the extent of ILS‐induced discordance across the genome should be reduced in the regions with high density of selection targets (Hobolth et al. 2011), unless *N*_e_ in the extant lineages differs significantly from one of the ancestral populations (Slatkin and Pollack 2006). Thus to estimate the extent to which ILS might have shaped the genome evolution in peatmoss, we investigated how phylogenetic discordance, a measure of ILS, is distributed across the 49 longest scaffolds in 100‐kbp sliding windows in relation to gene density, differentiation, divergence, and diversity.

**FIGURE 7:**
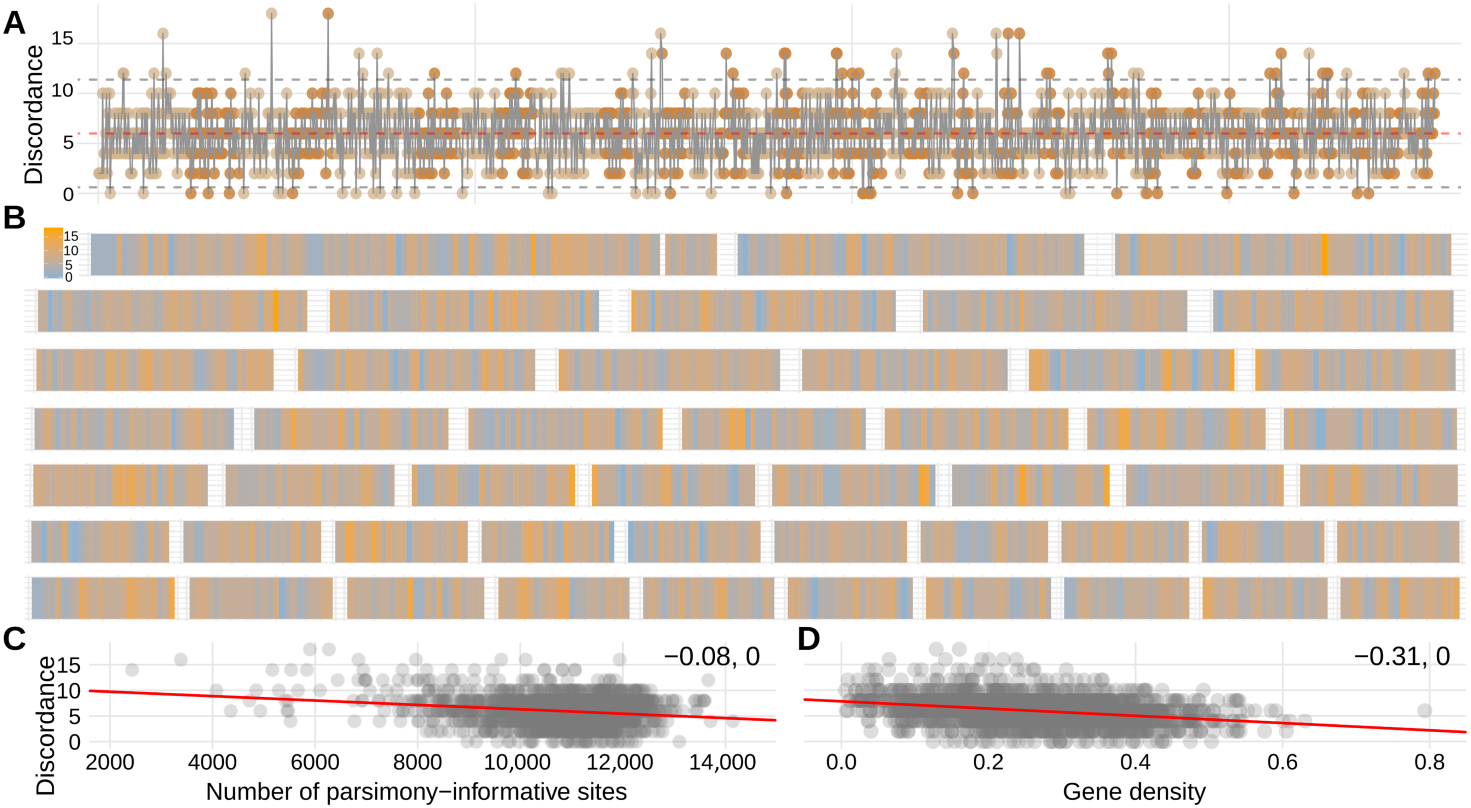
Topological discordance for sliding window trees across the 49 longest scaffolds. (A) Distribution of topological discordance score across the genome, the *x* axis corresponds to 100‐kbp sliding windows, colors refer to the scaffold the sliding window is located on. (B) Distribution of topological discordance score, colors refer to the value of topological discordance score in the corresponding sliding window. Relationships between the topological discordance score and (C) the number of parsimony‐informative sites, and (D) gene density per window, the numbers in the top right corner are Spearman's rank correlation coefficient followed by *P* value.

To characterize the distribution of incongruence across the genome, we assessed concordance between individual 100‐kbp sliding window trees and the species tree reconstructed by Meleshko et al. (2021). We kept one accession per species and calculated the topological distance score, equal to the number of steps required to interconvert the compared unrooted trees (Penny and Hendy 1985; Rzhetsky and Nei 1992). A high score of this metric represents a high mismatch between the species tree and a sliding window tree. The analysis showed that topological discordance was highly variable among and within the genomic scaffolds, ranging from 0 (the same topology as the species tree) to 18 (5.99 ± 2.70 SD, Figure 7B). Most of the scaffolds contain outliers that differ from the genome‐wide mean discordance score by >2 SD, showing very low correspondence to the species tree (Figure 7A, Figure S6). There was only a weak negative correlation between topological discordance and the number of parsimony‐informative sites in the alignment (*r*_S_ = −0.08, *p* < 0.0001, Figure 7C). Therefore, variable discordance across the genome is real and not of technical origin.

We found that windows with lower topological discordance have significantly higher gene density (*r*_S_ = −0.31, *p* < 0.0001, Figures 4 and 7D). Moreover, windows with lower topological discordance have significantly higher differentiation (*F*_ST_), and lower 𝜋 and *d*_XY_ (*r*_S_ = −0.27, 0.31, and 0.3, respectively, *p* < 0.0001, Figure 4). Such local increase in *F*_ST_ and phylogenetic concordance may stem from rapid sorting of ancestral variants due to reduced diversity, resulting from recurrent linked selection (e.g., background selection or selective sweeps) (Pease and Hahn 2013; Stankowski et al. 2019). Directional selection would lead to a wider span of concordance at linked sites (Slatkin and Pollack 2006), thus under directional selection one would expect spatial aggregation of regions with high concordance over longer distances than under the neutral model involving ILS (Slatkin and Pollack 2006; Hobolth et al. 2011). Therefore, we tested if topological incongruence was spatially aggregated across the genome by performing autocorrelation analysis along the scaffolds using a lag size of 3–10 corresponding to 0.3–1 Mbp (Figure S7). We found that 84%–98% of the windows did not demonstrate significant correlation with the neighboring windows (*p* < 0.01, Figure S8) implying that topological discordance was spatially aggregated at this scale in only a few cases, mostly at the lag size of 0.3 Mbp (see autocorrelation analysis in ‘Section 4’). The window sets with significant and insignificant autocorrelation coefficients did not differ from each other in discordance scores for all lag sizes (Student's *t*‐test, Figure S9).

## 3 Discussion

In this study, we found high correlations among genomic landscapes of genetic diversity (𝜋), divergence (*d*_XY_), and differentiation (*F*_ST_) of 12 peatmoss species which suggests that genome evolution in these species has been constrained by distribution of selection targets in the genome. The analyses of correlation and spatial co‐distribution of the statistics across the genome showed that regions with higher gene density have higher genetic differentiation, lower divergence, and nucleotide diversity, as well as higher number of rare alleles and lower phylogenetic discordance. Our analyses of sympatric and allopatric species pairs rule out gene flow as a major factor affecting the diversification landscape. Taken together, our findings indicate that the diversification landscapes in the studied species have been shaped by recurrent linked selection constrained by distribution of functional elements in the genome. Below, we discuss how these and other factors may have influenced genome evolution during speciation in peatmosses.

### 3.1 Correlated Landscapes of Differentiation Suggest Constrained Genome Evolution

Multiple studies report conserved diversification landscapes in various species across the speciation continuum. This is attributed to conserved distribution of functional features in the genome, such as gene density and/or recombination rate, which imposes restrictions onto the magnitude and genomic consequences of evolutionary forces acting on the genomes of diversifying lineages, resulting in evolution of similar genomic landscapes of divergence and diversity in them (Martin et al. 2013; Irwin et al. 2016; Vijay et al. 2016; Delmore et al. 2018; Stankowski et al. 2019; Chase, Ellegren, and Mugal 2021; Liang et al. 2022). Our findings of highly correlated landscapes of differentiation described here suggest that peatmosses join the growing number of animal and plant groups where conserved distribution of functional genomic features condition genome evolution under speciation.

### 3.2 The Role of Linked Selection in Shaping the Genomic Landscape of Peatmoss Diversification

Haploid nature and even distribution of selection targets across the genome suggest that direct and linked effect of positive and/or purifying selection is expected to be strong in peatmoss genomes. Indeed, we argue that the diversification landscape observed in the studied species can be best explained by the effect of recurrent linked selection. In particular, the covariation among measures of genetic diversity, phylogenetic discordance, and gene density, as demonstrated here, is a predicted consequence of long‐term purifying selection and/or background selection (BGS) (Burri 2017). Cumulative indirect effects of recurrent BGS largely explain the genome‐wide variation in genetic diversity and correlated landscapes of differentiation in many organisms, especially at late stages of differentiation (Burri 2017; Rettelbach, Nater, and Ellegren 2019). BGS also better explains the negative correlation between divergence and differentiation since, in outcrossing sexual organisms, it affects *d*_XY_ more strongly than *F*_ST_ (Matthey‐Doret and Whitlock 2019). Similarly, the relationships observed between genetic diversity, gene density, and Tajima's *D* can be explained by the effect of recurrent BGS/purifying selection. Low Tajima's *D* could also indicate recent population expansion leading to excess of rare variants (Tajima 1989), but it would not be expected to be associated with regions of higher gene density (Hahn, Rausher, and Cunningham 2002; Stajich and Hahn 2005), in contrast to our observations. Finally, the genomic landscapes of Tajima's *D* and diversity strongly resemble those produced in simulations under the BGS scenario in *Drosophila* whose genome has a similar gene‐dense structure (Schrider 2020), which further substantiates the predominant effect of BGS.

### 3.3 Incomplete Lineage Sorting Is Widespread in the Genome but Is Modulated by Selection Targets Density Variation

Peatmosses are characterized by extensive ILS (Meleshko et al. 2021). Recurrently, acting selection reduces neutral genetic variation and *N*_e_, and thereby facilitates lineage sorting at linked sites (Pollard et al. 2006; Hobolth et al. 2011; Pease and Hahn 2013; Li et al. 2019). This effect depends on the strength of linkage and is more pronounced in the regions with higher density of selection targets (Kaplan, Hudson, and Langley 1989; Hudson and Kaplan 1995; Cutter and Payseur 2013; Schrider 2020). Accordingly, we find that phylogenetic discordance is heterogeneously distributed across the genome and negatively, albeit not strongly, associated with gene density. Under recurrent linked selection at late stages of speciation, however, stronger correlation is expected, unless the species evolved considerable differences in recombination rate or gene conversion occurred in the regions of low recombination (Pollard et al. 2006; Burri 2017). The latter is not the case since we do observe correlated diversity, divergence, and differentiation landscapes among the species, which would only be possible with conserved recombination landscape (reviewed in Burri 2017). It has been suggested that under the neutral model with ILS, regions harboring loci responsible for establishment of reproductive isolation between species via hybrid incompatibilities, that is, speciation genes, are expected to produce evolutionary relationships discordant with the species trees (Wang and Hahn 2018). This could explain why discordance was quite strong even in regions with high gene density. The genetic mechanisms and the extent of reproductive isolation in peatmosses are unknown; therefore, this should be interpreted with caution. In addition, peatmosses are characterized by large ancestral *N*_e_ (Stenøien and Såstad 1999; Szövényi et al. 2008; Yousefi et al. 2017) and long generation times, which are expected to increase ILS even further (Copetti et al. 2017).

### 3.4 Effects of Demographic History

Natural selection is expected to affect much smaller, localized regions of the genome in comparison with the more broad, genome‐wide effects of demographic events (Stajich and Hahn 2005; Schrider 2020). The combination of recurrent selection coupled with a recent bottleneck has been shown to have strong effects on the genome in many organisms, including humans (Stajich and Hahn 2005), crucifers (Slotte et al. 2010), monkeyflowers (Stankowski et al. 2019), and poplar species (Ma et al. 2018), among others. The Northern Hemisphere peatmoss species have experienced massive bottlenecks during the Last Glacial Maximum (reviewed in Kyrkjeeide et al. 2014). This entails numerous consequences for the subsequent evolutionary history of the studied species and complicates the interpretation of the results of our analyses. First, contemporary *N*_e_ may differ markedly from ancestral population sizes, resulting in deviation of the expected relationships between phylogenetic discordance and functional features distribution (Slatkin and Pollack 2006). Secondly, bottleneck events followed by population expansion can lead to false signatures of selection in the genome, and, in particular, in patterns resembling selective sweeps instead of correctly inferring BGS (reviewed in Schrider 2020). Nevertheless, fluctuations in effective population size do not change the fact that recurrent linked selection is expected to produce patterns of genetic diversity and differentiation deviant from that expected under the neutral model (Zeng 2013).

## 4 Conclusions

In this study, we demonstrate that genome evolution in the studied species has mainly been shaped by long‐term effects of linked, most likely purifying, selection, constrained by distribution of selection targets in the genome. Future perspectives could involve comparing the differentiation landscapes in multiple species according to the time since their split, while taking into account the demographic history of each species.

Our findings are in line with studies in vascular plants, for example, oaks (Liang et al. 2022), sunflowers (Renaut et al. 2013), poplar (Wang et al. 2020), and *Primulina* (Ke et al. 2022) and therefore suggest that genome evolution is guided by similar processes in bryophytes and vascular land plants. Speciation on genomic level is primarily studied in angiosperms, whereas our study demonstrates that bryophytes hold a great potential for this line of research, calling for facilitating the expansion of the genomic resources in this remarkable group of plants.

## 5 Materials and Methods

### 5.1 Dataset

We used whole‐genome resequencing data obtained in the previous study (Meleshko et al. 2021) comprising 190 individuals from 12 haploid co‐occurring peatmoss species (Table S1) and sampled from sympatric/parapatric and allopatric populations at three geographical scales in Europe (Table 1). Sequencing data processing, read mapping, and filtering was performed using a reference genome assembly of *Sphagnum angustifolium* v0.5 (Healey et al. 2023) and the Paleomix pipeline v1.2.13.4 (Schubert et al. 2014) as described in (Meleshko et al. 2021).

### 5.2 Population Genomic Analyses

We used ANGSD v0.931 (Korneliussen, Albrechtsen, and Nielsen 2014) to calculate nucleotide diversity and neutrality statistics within species, as well as species differentiation based on site frequency spectrum (SFS), without calling individual genotypes. First, we performed quality filtering of mapped reads, which we hereafter refer to as “reads quality filtering.” We carried out per‐base alignment quality (BAQ) computation (Li 2011) implemented in ANGSD, and adjusted mapping quality score (MAPQ) to 50 for reads containing excessive mismatches. We discarded secondary alignments and reads with unmapped mate, poor quality (flag ≥ 256), low MAPQ score (≤30), or low base quality score (≤20). We discarded individuals from a site if individual filtered read depth at that site differed from 2 to 100.

We further used ANGSD to estimate SFS based on genotype likelihoods (GL) computed using the SAMTools method (Li et al. 2009) setting ploidy level to 1 (*‐isHap 1*). Based on GLs in biallelic sites, we calculated allele frequencies and inferred minor alleles using ML approach (Skotte, Korneliussen, and Albrechtsen 2013). We discarded sites missing in more than ⅓ of individuals. Then, site allele frequency likelihood (SAF) was estimated jointly for all individuals for each species. We also estimated the SAF for each population. Using the expectation maximization (EM) algorithm, we optimized and polarized the SAF to obtain an ML estimate of the SFS for each species (Nielsen et al. 2012) and an ML estimate of the pairwise (2D) SFS for each species pair.

We used the Empirical Bayes method implemented in ANGSD to perform neutrality test statistics and to calculate *F*_ST_ in sliding windows across the genome, using the inferred SFS to take into account genotyping uncertainty (Korneliussen et al. 2013; Fumagalli et al. 2013). Instead of performing computationally intensive estimation of an ML SFS for each sliding window, the method calculates posterior probabilities for the SFS at each site using a prior. For neutrality test statistics, the prior is the joint ML estimate of the SFS for the whole genome generated at the previous step (Korneliussen et al. 2013). Without inferring derived alleles, the posterior estimates of 𝛳_W_ and 𝛳_π_ are obtained for each window as linear functions of the folded ML estimate of the SFS assuming the infinite size model, and Tajima's *D* is calculated for each window assuming neutral model without recombination as $T=\left(\mathrm{ϴ\pi}-\mathrm{ϴW}\right)/\sqrt{\mathrm{var}\left(\mathrm{ϴ\pi}-\mathrm{ϴW}\right)\;}$ (Korneliussen et al. 2013) SFS for each species pair (Fumagalli et al. 2013). Using the folded estimate of 2D SFS, we calculated whole‐genome weighted *F*_ST_ for each species pair, as well as in sliding windows, in ANGSD (Fumagalli et al. 2013) using an extended version of the method‐of‐moments estimator (Reynolds, Weir, and Cockerham 1983). We used 100‐kbp sliding nonoverlapping windows and kept the scaffolds longer than 2 M and 1 M bases that equal to 44.3% (175.6 M bases) and to 70.3% (278.6) of the total length of the reference for the sliding‐window based estimates and the whole‐genome *F*_ST_ calculation, respectively. The cutoff of the scaffolds length was chosen to maximize the number of windows per scaffold and exclude scaffolds with low quality, that is, with high repeat content and contamination, and low number of gene models. We calculated correlation between pairwise *F*_ST_ matrix and geographical distance matrix using a Mantel test (Mantel 1967) within the package “ape” (Paradis and Schliep 2019).

To estimate divergence (*d*_XY_) among the species, we used the collection of scripts for genomic data analysis by Martin (https://github.com/simonhmartin/genomics_general). We calculated *d*_XY_ among all species in 100‐kbp consecutive windows using the script popgenWindows.py with command line argument “popDist” based on nuclear genome consensus fasta alignments for each sliding window generated using ANGSD. To obtain the alignments, we performed read quality filtering on mapped reads and used the filtered reads to generate fasta files for each sample in ANGSD. At each position, we sampled the base with the highest effective base depth (EBD), which is a product of mapping quality and base quality scores for each base. This method offers better precision in base calling from low‐coverage sequencing data (Wang et al. 2013). We then used a custom bash script and SeqKit (Shen et al. 2016) to slice the individual alignments into 100‐kbp consecutive windows and convert them into multiple sequence alignment fasta files for each sliding window. We discarded one sample (UH58) based on the high number of missing bases. To verify that the exclusion of UH58 did not affect the statistics estimates, and to compare the statistics calculated based on GLs and on the alignments, we further used popgenWindows.py to calculate 𝜋 and *F*_ST_. The mean window‐based values for each statistic were significantly highly correlated to the ones obtained based on GLs in ANGSD (*r*_S_ = 0.98 for 𝜋, *r*_S_ = 0.96 for *F*_ST_, *p* < 0.0001). We used GLs‐based 𝜋 and *F*_ST_ for all subsequent analyses described below.

We visually inspected the genomic distribution of these sliding‐window statistics for all species or pairwise comparisons concluding that the genomic landscapes of within‐species diversity (𝛳_π_, hereafter referred to as 𝜋) and Tajima's *D*, and between‐species differentiation (*F*_ST_) and divergence (*d*_XY_) were correlated across all species. Thus, we performed PCA on the sliding‐window data to summarize the across‐species variation in these statistics. The variation explained by the principal component can be used as a proxy for the degree of the correlation among the genomic landscapes across the species (Burri 2017). We extracted the loadings of each sliding window onto PC1 and calculated their correlation with the mean interspecific window‐based values for each statistic. We also obtained Spearman's correlation coefficient between PC1 for a statistic and the corresponding window‐based values for all the pairwise comparisons. False discovery rate correction was performed for these and described below correlation analyses using Benjamini and Hochberg correction (Benjamini and Hochberg 1995).

### 5.3 Phylogenetic Discordance

To estimate the distribution of phylogenetic concordance across the genome, we used the consensus alignments prepared as described above to generate phylogenetic trees in 100‐kbp consecutive sliding windows. We used IQ‐TREE v1.6.12 (Nguyen et al. 2015) to infer the best ML tree for each window using GTRGAMMA+I model and 1000 ultrafast bootstrap replicates (Hoang et al. 2018). We then calculated a measure of phylogenetic discordance between the estimated consensus sliding window trees and the species tree reconstructed from the same dataset by Meleshko et al. (2021) using a topological distance score in the package “ape.” The score is the partition metric defined as the number of steps required to transform the compared unrooted trees into one another (Penny and Hendy 1985; Rzhetsky and Nei 1992). We kept one sample per species in the sliding window trees and the species tree as branching order within each species was poorly resolved in the species tree, whereas all species were well‐resolved as monophyletic clades.

### 5.4 Correlation Analysis

We calculated Spearman's correlation coefficient between the phylogenetic discordance score and gene density, gene count, and population genetic parameters such as 𝜋, Tajima's *D*, *d*_XY_, and *F*_ST_. Gene density was estimated as a ratio of the total number of bases within all gene regions located in a given sliding window to the total length of that window, whereas gene count corresponds to the number of genes located in the sliding window. We also calculated spatial autocorrelation coefficients along the scaffolds for phylogenetic discordance score, 𝜋, Tajima's *D*, *d*_XY_, and *F*_ST_. For each statistic, we calculated Spearman's correlation between the adjacent window sets using lag size of 3–10 windows corresponding to 0.3–1 M bases. The significance of autocorrelation coefficients was determined from a null distribution of the autocorrelation values obtained from 1000 random permutations of the genome‐wide data. The results of this and other analyses were visualized using the package “lattice” (Sarkar 2008), “corrplot” (Wei and Simko 2017), and “ggplot2” (Wickham 2016) in the R statistical environment v3.6.3 (R Core Team 2020).

## Conflicts of Interest

The authors declare no conflicts of interest.

## Supporting information


**Figure S1.** Principal component analysis of pairwise *F*_ST_ and divergence in 100 Kb sliding windows for all species comparisons.
**Figure S2.** Within‐species diversity and Tajima’s *D*.
**Figure S3.** Correlated genomic landscapes of summary statistics across 49 biggest scaffolds.
**Figure S4.** Gene density distribution.
**Figure S5.** Principal component analysis of difference between sympatric and allopatric pairwise *F*_ST_ in 100 Kb sliding windows for all species comparisons.
**Figure S6.** Phylogenetic discordance score in 100‐kbp sliding window trees summarized for each of the 49 longest scaffolds.
**Figure S7.** Autocorrelation of phylogenetic discordance.
**Figure S8.** Summary of autocorrelation analysis for all statistics analysed.
**Figure S9.** Mean phylogenetic discordance in windows sets of different lag sizes (x axis) with significant and insignificant autocorrelation coefficients.
**Table S1.** List of The European Nucleotide Archive sample accessions for all specimens used in the study.
**Table S2.** Summary of all summary statistics calculated accounting for among‐species diversity.

## Data Availability

The sequence data used in this study are available in The European Nucleotide Archive, and the accession numbers are provided in the Table S1.
