# Supplementary material for: Linked Selection and Gene Density Shape Genome‐Wide Patterns of Diversification in Peatmosses
Source: Evol Appl. 2024 Aug 19;17(8):e13767. doi: 10.1111/eva.13767 (PMC11333200; doi:10.1111/eva.13767)
Supplement: Supplementary file 1 — Figure S1. Principal component analysis of pairwise F ST and divergence in 100 Kb sliding windows for all species comparisons. Figure S2. Within‐species diversity and Tajima’s D. Figure S3. Correlated genomic landscapes of summary statistics across 49 biggest scaffolds. Figure S4. Gene density distribution. Figure S5. Principal component analysis of difference between sympatric and allopatric pairwise F ST in 100 Kb sliding windows for all species comparisons. Figure S6. Phylogenetic discordance score in 100‐kbp sliding window trees summarized for each of the 49 longest scaffolds. Figure S7. Autocorrelation of phylogenetic discordance. Figure S8. Summary of autocorrelation analysis for all statistics analysed. Figure S9. Mean phylogenetic discordance in windows sets of different lag sizes (x axis) with significant and insignificant autocorrelation coefficients. Table S1. List of The European Nucleotide Archive sample accessions for all specimens used in the study. Table S2. Summary of all summary statistics calculated accounting for among‐species diversity. [file EVA-17-e13767-s001.pdf]

Supplementary Materials

Table S1. Specimens used in the study. ENA - The European Nucleotide Archive. The specimens' tissues are deposited in Trondheim Herbarium, for accession IDs and detailed sequencing summary see Table S1 in Meleshko et al. 2021.

Species	Number of samples	ENA study accession	ENA sample accessions
Sphagnum capillifolium	17	PRJEB39751	SAMEA7502614-SAMEA7502630
Sphagnum compactum	13	PRJEB39751	SAMEA7502631-SAMEA7502643
Sphagnum divinum	18	PRJEB39751	SAMEA7502644-SAMEA7502661
Sphagnum fimbriatum	11	PRJEB39751	SAMEA7502662-SAMEA7502673
Sphagnum flexuosum	14	PRJEB39751	SAMEA7502674-SAMEA7502687
Sphagnum fuscum	19	PRJEB39751	SAMEA7502688-SAMEA7502706
Sphagnum lindbergii	14	PRJEB39751	SAMEA7502707-SAMEA7502720
Sphagnum platyphyllum	12	PRJEB39751	SAMEA7502721-SAMEA7502732
Sphagnum riparium	14	PRJEB39751	SAMEA7502733-SAMEA7502746
Sphagnum squarrosum	19	PRJEB39751	SAMEA7502747-SAMEA7502765
Sphagnum subnitens	19	PRJEB39751	SAMEA7502766-SAMEA7502784
Sphagnum tenellum	20	PRJEB39751	SAMEA7502785-SAMEA7502804

Table S2. Summary of all summary statistics calculated accounting for among-species diversity.

	Mean	Median	SD	SE	Coefficient of Variation	Minimum	Maximum	Upper Quantile 75%	Lower Quartile 25%	Sample size
Discordance	5.99	6.00	2.70	0.065	0.45	0.00	18.00	8.00	4.00	1729
Gene count	7.05	7.00	2.84	0.068	0.40	0.00	17.00	9.00	5.00	1729
Gene density	0.26	0.26	0.12	0.003	0.45	0.00	0.79	0.34	0.17	1729
Mean d_{XY}	0.02	0.02	0.0 02	0.000	0.08	0.02	0.03	0.02	0.02	1729
Mean F_{ST}	0.90	0.92	0.05	0.001	0.06	0.53	0.97	0.93	0.89	1728
Mean π	0.002	0.002	0.001	0.000	0.42	0.001	0.007	0.002	0.002	1728
Mean Tajima's D	-3.19	-3.32	0.45	0.011	-0.14	-3.97	-0.65	-3.02	-3.48	1729

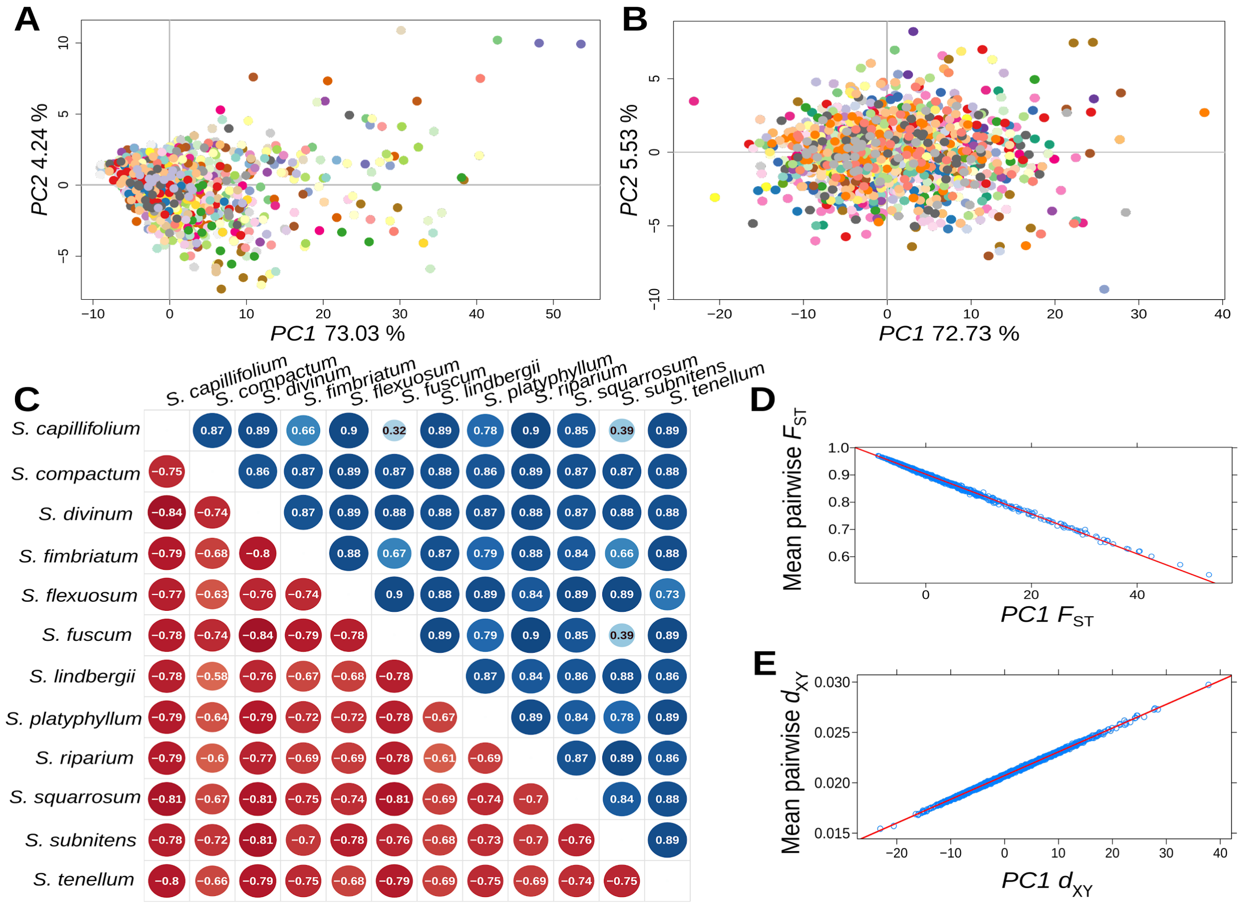

Fig. S1. Principal component analysis of pairwise F_{ST} and divergence in 100 Kb sliding windows for all species comparisons. (A) PCA plot of F_{ST}, (B) PCA of d_{XY} in 100-kbp sliding windows for all species, colour represents the scaffold the sliding window is located on, individual points correspond to the sliding windows, (C) Spearman's rank correlation coefficients between the loadings onto *PC1* and pairwise values of F_{ST} for all species (lower triangle) and d_{XY} for all species (upper triangle), all correlation was significant with $p<0.0001$, (D) Spearman's rank correlation coefficients between the loadings onto *PC1* and mean window-based values of pairwise F_{ST} across the species, (E) Spearman's rank correlation coefficients between the loadings onto *PC1* and mean window-based values of pairwise d_{XY} across the species.

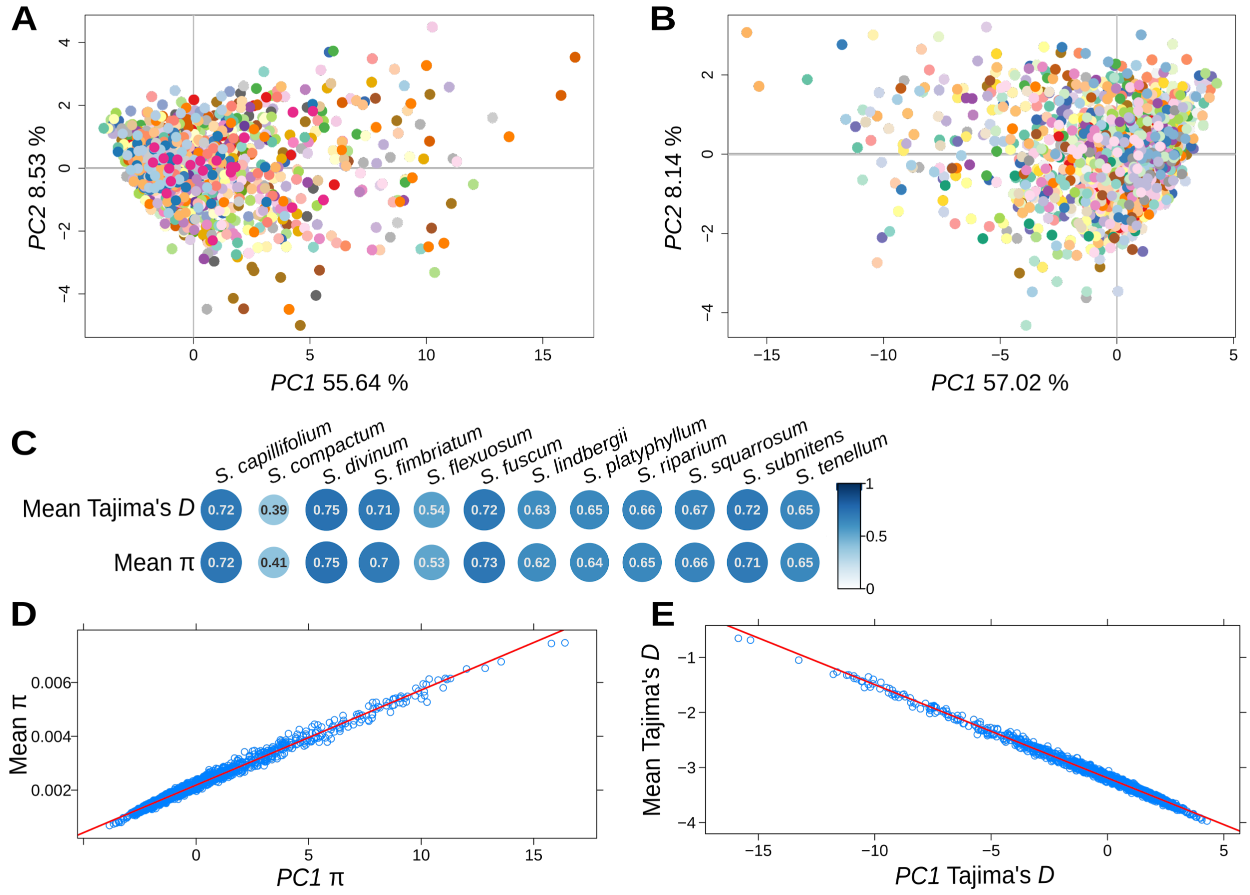

Fig. S2. Within-species diversity and Tajima's *D*. (A) PCA of π in 100-bp sliding windows for all species, (B) PCA for Tajima's *D* in 100-kbp sliding windows for all species, colour represents the scaffold the sliding window is located on, individual points correspond to the sliding windows, (C) Spearman's rank correlation coefficients between mean and within-species values of Tajima's *D* and π, (D) Spearman's rank correlation coefficients between the loadings onto *PC1* and mean π across the species, (E) Spearman's rank correlation coefficients between the loadings onto Tajima's *D PC1* and mean Tajima's *D* across the species.

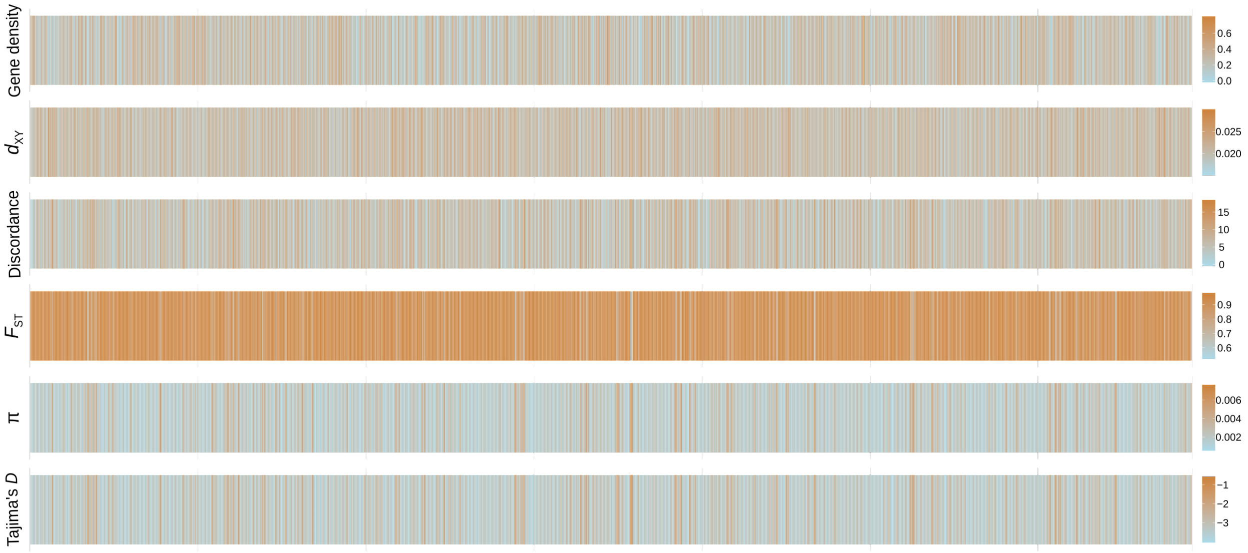

Fig. S3. Correlated genomic landscapes of summary statistics across 49 biggest scaffolds

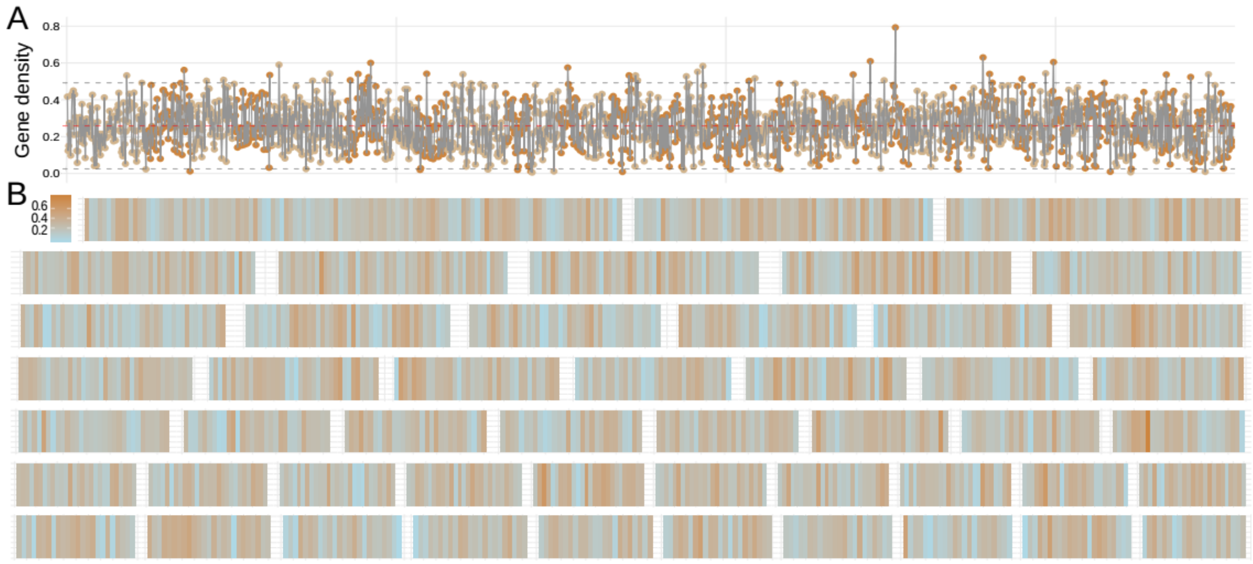

Fig. S4. Gene density distribution in 100-kbp sliding windows (A) across the 49 longest scaffolds, mean genome-wide density is shown in red, grey dotted lines represent mean genome-wide density±2SD, (B) in each scaffold, colours represent gene density as shown on the right.

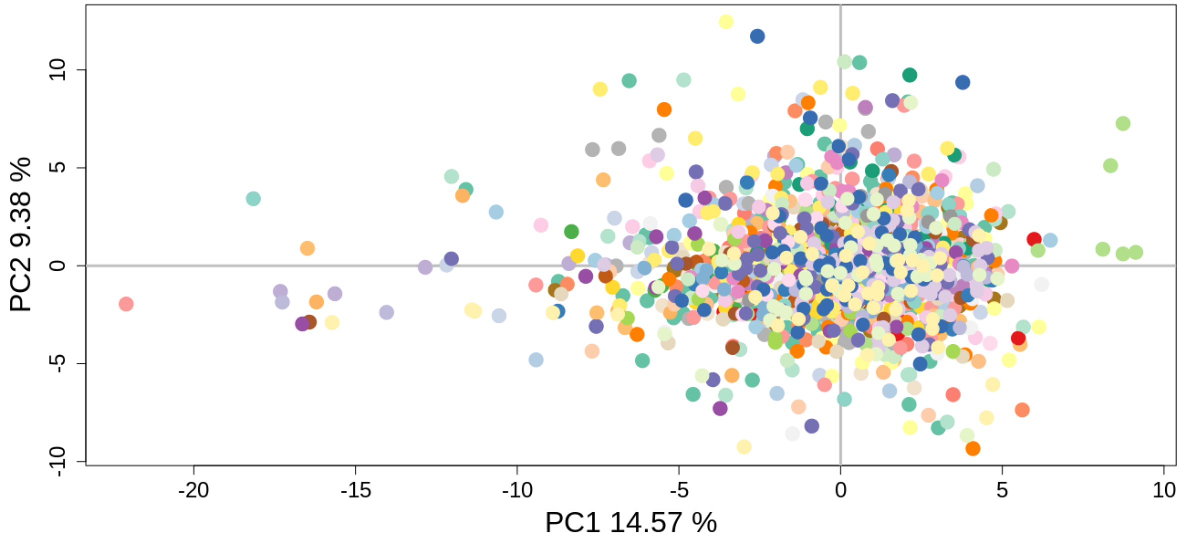

Fig. S5. Principal component analysis of difference between sympatric and allopatric pairwise F_{ST} in 100 Kb sliding windows for all species comparisons, colour represents the scaffold the sliding window is located on, individual points correspond to the sliding windows.

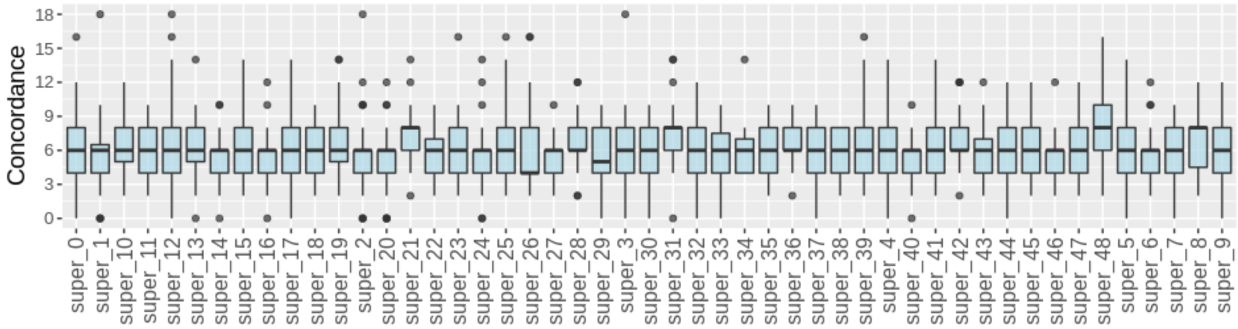

Fig. S6. Phylogenetic discordance score in 100-kbp sliding window trees summarized for each of the 49 longest scaffolds. In all boxplots, the central line is the median, and lower and upper hinges represent the first and third quartiles.

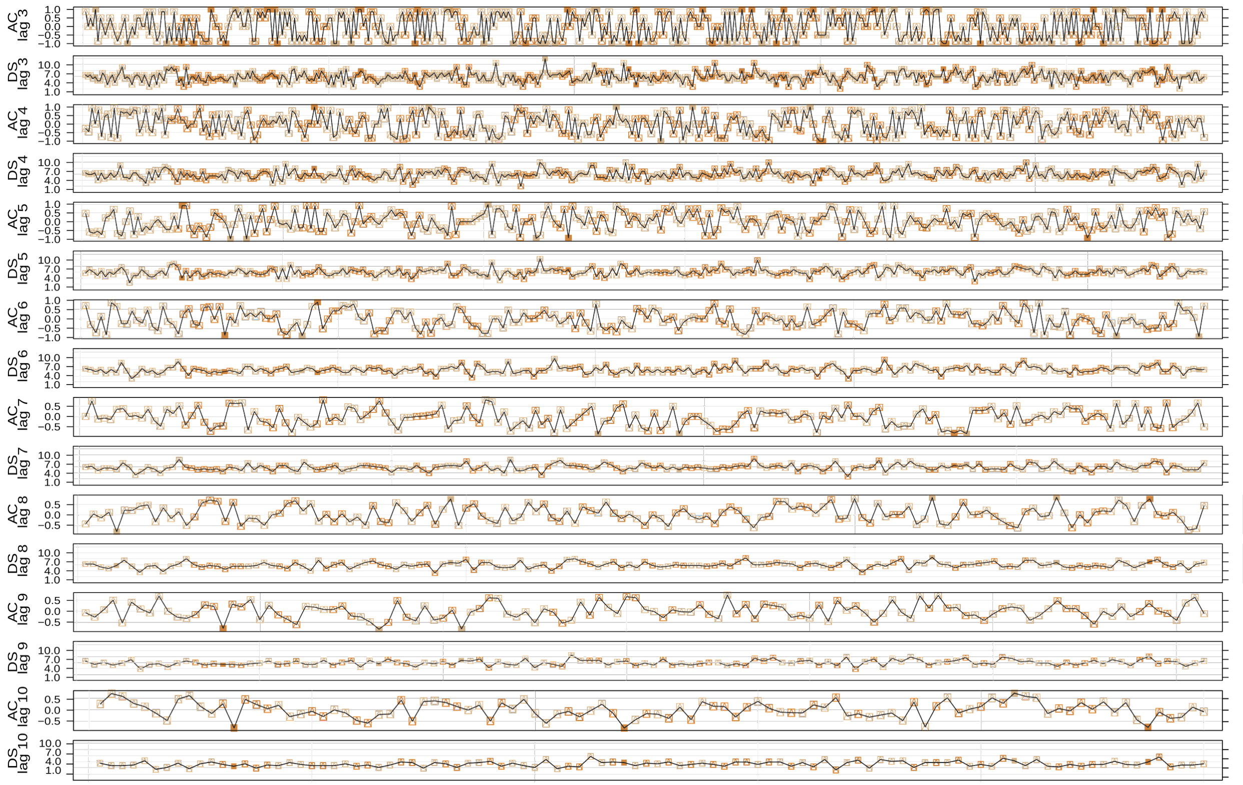

Fig. S7. Autocorrelation of phylogenetic discordance. The alternating panels show the distribution of autocorrelation coefficients (AC), and mean discordance scores (DS) in the corresponding set of windows across the genome, points filled with colour have a significant autocorrelation coefficient ($p<0.01$) with the next window set. Lag 3 to 10 correspond to lag sizes of 0,3 to 1 Mb ($p=0.01$), alternating colours represent different scaffolds.

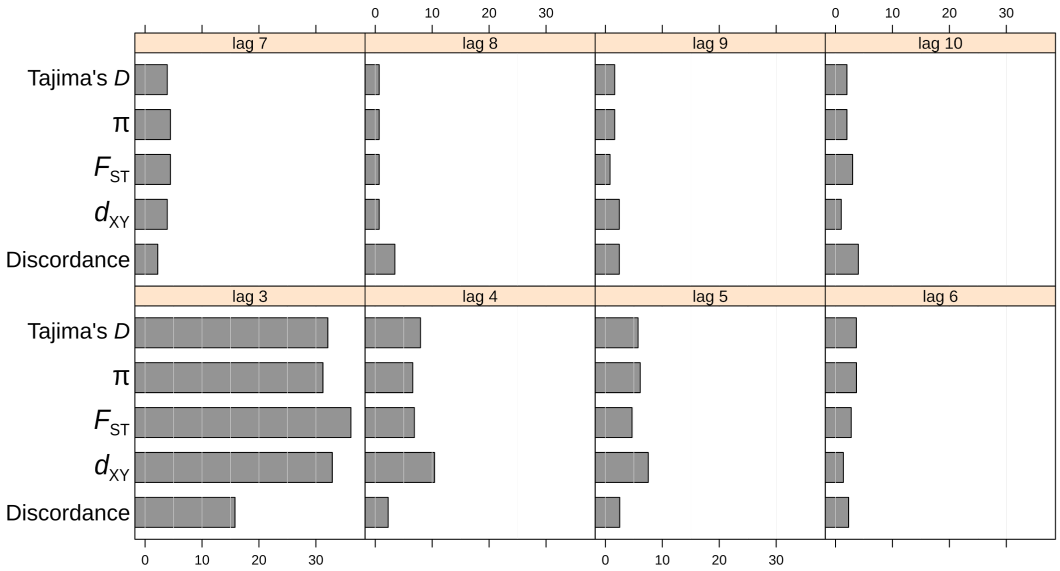

Fig. S8. Summary of autocorrelation analysis for all statistics analysed. The x axis shows the percentage of windows sets showing significant spatial correlation with the next window set for lag sizes of 0,3 to 1 Mb (p<0.01).

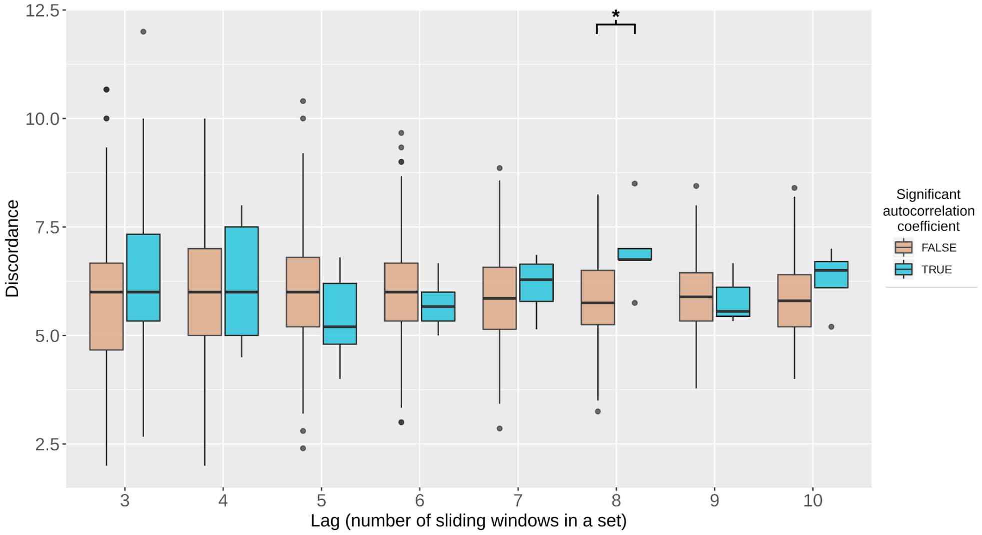

Fig. S9. Mean phylogenetic discordance in windows sets of different lag sizes (x axis) with significant and insignificant autocorrelation coefficients. Statistically significant comparisons are marked with * (Student's t-test, p=0.02). In all boxplots, the central line is the median, and lower and upper hinges represent the first and third quartiles.
